# Children’s Social Integration and Low Perception of Negative Relationships as Protectors Against Bullying and Cyberbullying

**DOI:** 10.3389/fpsyg.2019.00643

**Published:** 2019-03-22

**Authors:** Nieves Moyano, Ester Ayllón, José Luis Antoñanzas, Jacobo Cano

**Affiliations:** ^1^Facultad de Ciencias Humanas y de la Educación, Departamento de Psicología y Sociología, Universidad de Zaragoza, Huesca, Spain; ^2^Facultad de Educación, Departamento de Psicología y Sociología, Universidad de Zaragoza, Zaragoza, Spain; ^3^Facultad de Educación, Departamento de Ciencias de la Educación, Universidad de Zaragoza, Zaragoza, Spain

**Keywords:** coexistence, bullying, cyberbullying, social integration, negative relationships

## Abstract

The aim was to investigate the factors associated with the diverse bullying forms suffered by a victim (relational, aggressive and cyberbullying) by considering the mediating role of the quality of coexistence in school: social integration and perception about relationships among peers. We evaluated data about 42 schools (79.5% public) in a sample of 3,407 students (47.6% boys and 52.4% girls) from the Primary Education. The mediational analyses indicated that, to predict all the bullying forms, a greater sense of social integration and a perception of low negative relationships were mediators, and social integration was the factor that most strongly correlated with bullying, especially relational bullying. We found that the number of good friends and negative relationships together predicted social integration, and the school type predicted negative relationships and number of good friends. The implications for education programs and policy are discussed.

## Introduction

School bullying research has focused mainly on the analysis of risk factors (see Saarento et al., 2015; Moore et al., 2017; Zych et al., 2018). Therefore, protective factors have often been neglected. While risk factors are crucial for the design of prevention programs, the study of protective factors would help us to better provide intervention programs based on fostering supportive friendships and adjustment (Ttofi et al., 2014; Brendgen and Poulin, 2018). In this sense, some protective factors such as social integration and the quality of school coexistence are seen as a key to reduce school bullying (Méndez et al., 2017) and to prevent children from bullying and its consequences (Zych et al., 2017).

Research on bullying has often been approached by the ecological framework of Bronfenbrenner (1979) (Swearer et al., 2010; Hong and Espelage, 2012; Espelage, 2014). Evidence from this model indicates a high explained variance for bullying behaviors (Lee, 2011). On the other hand, and due to recent evidence which highlights that the quality of relationships plays a relevant role in school bullying, other theoretical models have been provided. In particular, the “Model for Building Coexistence” integrates diverse mechanisms to facilitate coexistence and the quality of relationships by focusing on students (Córdoba et al., 2016; Ortega-Ruiz and Córdoba, 2017). Together, both standpoints are useful for the purposes of the present study.

The ecological model is defined by several systems. However, we aimed to focus on the microsystem level as this level comprehends the most proximal factors to bullying: individual and school environment factors, of which the latter are of relevance for both theoretical models. In particular, we focus on individual factors such as gender and age, and parents-related variables such as their education and nationality. On the other hand, school environment factors such as their social integration, friendships, and perceptions toward positive or negative interactions among peers are included. For example, previous research on individual’s variables indicate that boys are more often victims (Cook et al., 2010) but this depends on the bullying type (Baldry et al., 2017). Regarding age, in Spain the stronger school violence lies between 11 and 13 years (46% of the reported cases) (ANAR Foundation, 2016). Considering parent’s factors, level of education is associated with their children more likely being victims (Von Marées and Petermann, 2010). Parents’ nationality is also relevant. Previous studies show that the children who belong to an ethnic minority are more likely to be victimized (Vervoort et al., 2010; Rhee et al., 2017). In line with this, immigrant parents face some challenges such as adjustment to a different culture and promoting their children’s integration (Pottie et al., 2015). This may be the reason why some immigrant parents may feel that it is more difficult to take part in educational activities (Delgado-Gaitan, 1991; Waasdorp et al., 2011), probably due to language barriers, cultural values or education which can, in some ways, be linked to the disconnection and isolation of their children in the school system to a certain extent.

School environment is relevant for children and adolescents’ socialization, where their psychosocial adjustment is built (Sigfusdottir et al., 2016). Schools are sometimes a hostile place for students who, when they do not feel part of it, are likely to suffer from the passivity of school (Molina and Vecina, 2015; Martínez-Otero, 2017). Therefore, friendships –often measured by the number of good friends- has been shown as a protective factor against diverse physical, verbal and relational manifestations of bullying, but its relationship with cyberbullying is not clear (Cerezo et al., 2018; Konishi et al., 2018). Thus, students who feel more integrated in their education community tend to report lower school violence because they believe they form part of their school and feel less threatened by violence and bullying (Gendron et al., 2011; Klein et al., 2012). These aspects may also be influenced by other socio-demographic aspects linked to school, such as the number of years/courses enrolled in the same school is considered a facilitator for social integration. Students who have spent less time at the same school, that is, new students, are often the target of interventions to reduce bullying, as they are outside the social network and at the bottom of the power hierarchy (Mehta et al., 2013).

Our study aimed to make the following contributions: (a) Previous research has focused mostly to separately analyze relational bullying, aggressive bullying and cyberbullying. Therefore, an integrative study in which all these different forms of bullying are analyzed is needed; (b) It is likely that both protective factors and the absence of some risk factors play an important role in the decline of bullying, and that this phenomenon could be better examined by the interdependent associations between individual and contextual factors (see Espelage, 2014). Therefore, based on the review about school bullying in adolescents (Álvarez-García et al., 2015), the authors indicate these four types of predictors for being a bully: individual, family, school and community. Thus our study combines the variables from these main indicators: individual (gender, age); ethnicity, family (education, employment), school (climate, coexistence) and community (number of good friends), among others; (c) school environment factors concerning climate are documented to play a mediational role (Acosta et al., 2018). Thus our statistical approach to better understand diverse bullying forms was a mediational analysis (Hayes, 2012).

Recent research conducted across several countries indicates that the bullying phenomenon has declines somewhat in the last few years, probably due to the fruitful effects of intervention programs (Finkelhor, 2013; Zych et al., 2018). However, the reasons underlying this remain unknown. Therefore, the goal of the present study was to examine the following predictors: (a) the individual factors (gender, age, parents’ education and nationality); (b) socio-demographic school-related factors (number of courses enrolled at school and number of good friends). For this model, we investigated the mediating role of school-environment factors, such as quality of coexistence at school, measured by the sense of social integration and perception about positive or negative relationships among peers. Diverse bullying forms suffered by victims were taken as the outcome variables, namely relational or indirect, physical aggression or direct and cyberbullying.

## Materials and Methods

### Participants

We collected data from students from 42 schools in the Autonomous Community of Aragon (Spain). In this Autonomous Community, bullying rises to 13.4%, the fifth highest in Spain (PISA report, 2015). We recruited data from 3,490 boys and girls. After eliminating some cases in which individuals did not complete at least 75% of the survey, or due to some technical problems while filling them in, we examined data from 3,407 participants (47.5% boys, 52.4% girls) whose mean age was 11.04 (*SD* = 0.83). As seen in Table 1, as indicated by most participants, both the parents’ nationality was Spanish (80.2%) and with a high education, as 71.3% of fathers and 50.9% of mothers had a university degree. Approximately 89.6% indicated having 4–5 good friends at school. Regarding quality of coexistence, overall the students felt that they were socially integrated and they perceived relationships more positively than negatively. The frequency they reported having been bullied was low, and values came close to the lower limit of the range of scores.

**Table 1:** The socio-demographic characteristics of the sample (*N* = 3,407).

	*n* (%) / *M (SD)*
Gender	
Boys	1.622 (47.6)
Girls	1.785 (52.4)
Age	11.04 (0.83)
Course	
Year-5 Primary	1.717 (50.4)
Year-6 Primary	1.690 (49.6)
School type	
Public	2.708 (79.5)
State-funded school	699 (20.5)
Parents’ nationality	
Both Spanish	2.731 (80.2)
One Spanish	75 (2.2)
Neither was Spanish	601 (17.6)
No. of courses enrolled at school	7.43 (2.42)
Father’s education	
University degree	2.431 (71.3)
None – Secondary	976 (28.6)
Mother’s education	
University degree	1.039 (50.9)
None – Secondary	1.033 (49.1)
No. of good friends at school	
None	97 (2.8)
1–2	82 (2.4)
3–4	176 (5.2)
4–5	3.052 (89.6)
Quality of coexistence	
Social integration (range 3–12)	10.53 (1.62)
Perception of positive relationships	
(range 3–12)	8.03 (1.51)
Perception of negative relationships (range 3–12)	6.25 (2.40)
Bullying	
Relational (range 6–24)	7.11 (2.22)
Aggressive (range 6–24)	6.31 (1.17)
Cyberbullying (range 7–28)	7.28 (1.15)


### Measurements

- A socio-demographic background questionnaire with questions about gender, age, course, Spanish nationality (both parents, one parent or none, respectively, with 1, 2, and 3), level of education, school type (public or state-funded school) and number of good friends at school (from 0 to 4–5 friends).

- Quality of coexistence was measured by three components: Social integration and perceptions of relationships among peers, both positive and negative. It comprises nine items previously used in national studies, supported by the Spanish Ministry of Education about school coexistence and bullying (Díaz-Aguado et al., 2010). Three of the nine items were about social integration: “I easily make friends,” perception of positive relationships or relationships based on cooperation:“students help each other, but are not friends” (three items) and perception of negative relationships or conflicts (3 items): “fights occur among students.” The scores from these three items should be inverted to obtain an overall score by summing all the item scores. The answer scale ranged from 1 to 4, with 1 meant “completely disagree” and 4 denoted “completely agree.” Higher scores indicated greater social integration and better quality coexistence at school. We performed a confirmatory factor analysis (CFA) to examine the factorial structure. We obtained optimum values by the goodness-of-fit indices: χ^2^/*df* = 4.75; *p* = 0.000; RMSEA = 0.033; GFI = 0.99; TLI = 0.97. Cronbach’s alpha for this study was 0.69, with 69, 0.56, and 0.70, respectively, for each subscale: social integration, positive perception of relationships and negative perception of relationships.

- Bullying and Cyberbullying. In order to measure student bullying, we administered the self-reported measure previously used by Díaz-Aguado et al. (2013). The instructions for this measure indicate: Think whether you have suffered any of the following situations and mark the frequency you have suffered it in the last 2 months. It comprised 19 items answered on a 4-point Likert scale ranging from 1 (never) to 4 (many times). It provided scores for several bullying types: Relational Bullying: victims of situations of social exclusion or humiliation, measured by six items. For example “My schoolmates ignore me.” Physical Aggressive Bullying: it consists of six items that describe situations of aggression, such as “They hit me.” Cyberbullying: victims of violence related to social networks or technology; for example, Have any schoolmates recorded you by a mobile phone or video to go against you? (seven items). Although it goes beyond the scope of the present study, as no previous research has provided evidence for content validity regarding this three-factor structure, we performed a CFA with our data. The goodness-of-fit indices were adequate: χ^2^/*df* = 24.55, *p* = 0.000; RMSEA: 0.08; GFI: 0.88; TLI: 0.84. In order to improve these values, the modification indices indicated correlating errors from some of the items belonging to the same factor. However, no indications about either changes in items to another factor or eliminating any items that would jeopardize the theoretical subcomponents, were yielded by the analysis. Therefore, we completed this psychometric analysis by examining the internal consistency of the subscales. Cronbach’s alphas for all three bullying subtypes were 0.85, 0.77, and 0.76, respectively.

### Procedure

From the Regional Government of Aragon, diverse schools were selected by quota convenience sampling in the Autonomous Community of Aragon. An invitation letter was sent to schools to collaborate, in which information on the main study goals and the need for parental authorization and informed consent was established. A timeline reflecting the research phases was attached. Data were collected from March to April 2018 with the collaboration of the Principal and teachers. This phase was coordinated and supervised by research team members, who were working in each city/town by keeping in touch with each school personally and by telephone. Once consent from each school was confirmed, the schools to take part in the study received each user’s code and passwords to access the online survey. The students from each school completed the survey under similar conditions during school time in a laboratory using computers and with privacy. Some teachers accompanied the students to support them. Anonymity and confidentiality were guaranteed. The survey was completed in approximately 25–40 min.

This study was carried out following the recommendations from the Council of the British Educational Research Association of the Ethical Guidelines for Educational Research (British Educational Research Association [BERA], 2011), given that in Spain there is no ethical committee in educational research. Even though approval by an Ethics Committee was not required as per applicable institutional and national guidelines, the protocol was approved by the academic doctoral committee in a session held on 2014. This academic committee belongs to the University of Zaragoza, and it is not exclusively an ethics committee, but a scientific evaluation committee which incorporates the review of data collection procedures, including the ethical dimension. Also, this research is part of the Aragon I Plan against School Bullying (Order ECD/715/2016), which also financially supported the project.

Regarding the information about the families, after the consent from the educational centers that made us able to carry out the activity of data collection during the class schedule, all the families were informed about the objectives of the study and the voluntariness of the participation. Therefore, written informed consent was obtained from the parents of all participants. Those who did not agree to participate were not evaluated. This procedure is appropriate when the data collection is conducted within the classroom schedule. Anonymity and confidentiality was assured. Finally, the centers who took part in the study received a report with the main findings. This procedure does not involve experimentation with students, but rather a collection of data for educational and research purposes.

### Statistical Analyses

We first conducted zero-order correlation analyses to examine the association among the variables. We also performed a mediation analysis (Hayes, 2012) following the recommendations of Walters and Mandracchia (2017). Therefore, causal order and direction were previously established, and the model was tested by a confirmatory model in which both the direct and indirect effects were examined. Several mediation analyses were conducted, in which the following predictor variables were included: gender, age, parents’ education and nationality, and also type of school (public or state-funded school, number of courses enrolled at school and number of good friends at school). We tested the mediation effect of the quality of the coexistence subcomponents: social integration, and positive and negative relationships among peers. As dependent variables, we included: relational bullying, aggressive bullying and cyberbullying. Analyses were carried out using the macro PROCESS (Preacher and Hayes, 2008) in SPSS, which allows several mediator factors to be simultaneously analyzed (Hayes, 2012). These analyses were performed by a bootstrap analysis with 5000 samples, and a 95% confidence interval. Finally, we performed *structural equation modeling* (SEM) to confirm the adjustment of the models. These analyses were conducted using Mplus v. 6.1 (Muthén and Muthén, 2010a).

## Results

First at all, zero-order correlation analyses were performed to examine the association among the measured variables (see Table 2). The strongest significant correlations were found between the subscales of quality of coexistence (social integration, positive and negative relationships) and the three bullying types. The correlation between negative relationships among peers and being victims of bullying was positive.

**Table 2:** The zero-order correlations among the examined variables.

	1	2	3	4	5	6	7	8	9	10	11	12	13	14
(1) Gender		0.01	0.03	0.01	-0.01	-0.00	0.02	0.02	-0.01	-0.06***	0.00	0.02	0.07***	0.04*
(2) Age			0.01	0.06***	-0.06***	0.04*	0.06**	-0.05**	0.01	0.05***	0.02	-0.01	0.01	-0.05**
(3) School type				0.15***	0.10***	0.14***	0.27***	-0.09***	0.04**	-0.01	-0.07***	-0.02	-0.02	-0.04**
(4) Parents’ nationality					0.29***	0.10***	0.25***	-0.21***	-0.21***	-0.04*	0.10***	-0.07***	-0.05***	-0.03
(5) No. courses enrolled						0.03	0.20***	-0.29***	0.12***	0.02	-0.00	-0.07***	-0.05**	-0.04*
(6) Father’s education							0.59***	-0.05***	0.03*	-0.02	0.01	-0.04*	-0.04*	-0.04*
(7) Mother’s education								-0.19***	0.15***	0.02	-0.06*	-0.06**	-0.06**	-0.05*
(8) No. good friends									-0.12***	-0.01	-0.01	0.08***	0.07***	0.06***
(9) Social integration										0.37***	-0.10***	-0.41***	-0.22***	-0.14***
(10) Positive relationships											-0.28***	-0.21***	-0.11***	-0.10***
(11) Negative relationships												0.23***	0.19***	0.14***
(12) Relational Bullying													0.65***	0.47***
(13) Aggressive Bullying														0.61***
(14) Cyberbullying														


*^∗∗∗^*p* < 0.001; ^∗∗^*p* < 0.01; and ^∗^*p* < 0.05.*

We conducted mediation analysis (Hayes, 2012), following recommendations from Walters and Mandracchia (2017). Therefore, causal order and direction were previously established, and the model was tested by a confirmatory model in which both direct and indirect effects were examined. Several mediation analyses were conducted in which the following predictor variables were included: gender, age, parent’s education and nationality, and also type of school (public or state-funded school, number of courses enrolled at school and number of good friends at school). We tested the mediation effect of all the quality of coexistence subcomponents: social integration, and the positive and negative relationships among peers. As dependent variables, we included: relational bullying, aggressive bullying and cyberbullying. Analyses were carried out using the macro PROCESS (Preacher and Hayes, 2008) in SPSS, which allows several mediator factors to be simultaneously analyzed (Hayes, 2012). These analyses were performed through the bootstrap analysis with 5000 samples, and a 95% confidence interval.

The variables that mediated the relationship between predictors and bullying were social integration and perception of negative relationships among peers. However, perception of positive relationships among peers did not enter the model as no significant relationship was found. As predictors, we found that the interaction between parents’ nationality and number of courses enrolled at school was significant for both social integration and negative relationships, and number of good friends for social integration and type of school for negative relationships. However, the coefficients related to the predictive value of the parents’ nationality + number of courses interaction on social integration and perceived negative relationships were, albeit significant, very low. So they were removed from the model. Thus social integration and perception of negative relationships were mediators for all bullying forms, with a stronger relationship of both factors for predicting relational bullying. The mediational model to predict relational bullying explained 20% of variance, while the model to predict aggressive bullying explained 8% of variance. Finally, the model to predict cyberbullying scarcely explained 4% of variance.

**FIGURE 1:**
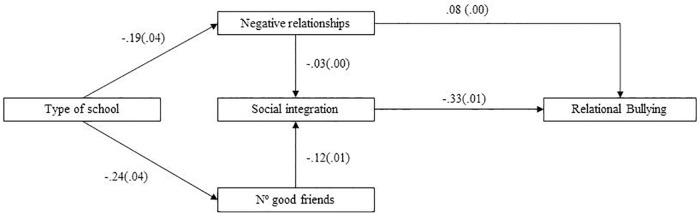
Structural equation modeling showing the standardized estimates between the predictor and mediational variables on Relational Bullying.

**FIGURE 2:**
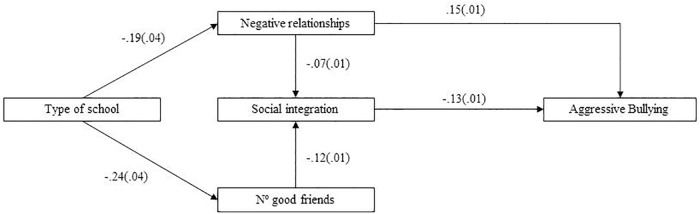
Structural equation modeling showing the standardized estimates between the predictor and mediational variables on Aggressive Bullying.

**FIGURE 3:**
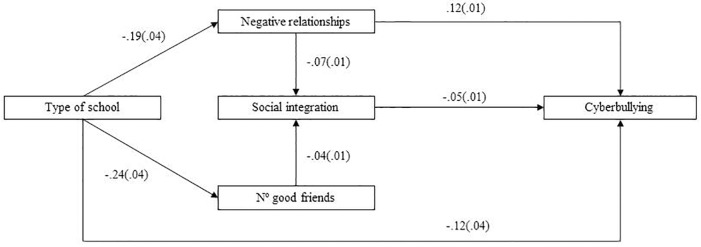
Structural equation modeling showing the standardized estimates between the predictor and mediational variables on Cyberbullying.

In order to test the adjustment of these three models, we performed SEM. We used the MLM, “the Maximum Likelihood Parameter estimates with standard errors and a mean-adjusted chi-square test statistic that are robust to non-normality. The MLM chi-square test statistic is also referred to as the Satorra-Bentler chi-square” (Muthén and Muthén, 2010b, p. 533). For the prediction of all forms of bullying, the modification indices suggested a direct effect of perception of negative relationships on social integration, in a negative direction. As Figure 1 depicts, in which all the standardized estimates are shown, for the Relational Bullying prediction, type of school predicted both negative relationships and number of good friends. In this sense, the route that comprises a larger number of good friends mediated by social integration feelings had a stronger impact on relational bullying, with a negative correlation than type of school mediated by negative relationships, which positively correlated with relational bullying. Figure 2 shows the model for Aggressive Bullying for which, once again, the route containing type of school, number of good friends and social integration had a stronger impact. However in this case, the standardized estimates were much lower than for the previous model. Finally as shown in Figure 3, for the Cyberbullying prediction, the standardized estimates from both routes were, albeit significant, very low, except for a direct effect shown by type of school on cyberbullying, with a negative direction. All three models were adjusted according to the goodness-of-fit indices. In particular, for Relational Bullying (χ^2^/*df* = 3.24, *p* = 0.000; RMSEA = 0.026; SRMR = 0.012; CFI = 0.98; TLI = 0.97), Aggressive Bullying (χ^2^/*df* = 3.64, *p* = 0.000; RMSEA = 0.028; SRMR = 0.015; CFI = 0.96; TLI = 0.90) and Cyberbullying (χ^2^/*df* = 2.72, *p* = 0.004; RMSEA = 0.023; SRMR = 0.009; CFI = 0.98; TLI = 0.91).

In short, in the three models, having more friends was associated with greater integration and students from state-funded school perceived fewer conflicts at school. For the mediators, feeling socially integrated and perceiving fewer conflicts lowered the likelihood of being bullied. However, it was interesting to note that perception of positive relationships did not enter any model; that is to say, perceiving positive relationships had little to do with being bullied or not.

## Discussion

School coexistence is a complex concept (Ortega, 2007; Pedrero, 2011; Peñalva-Vélez et al., 2015), in which both individual and interpersonal phenomena converge. As suggested by Peñalva and Soriano (2011), nowadays reach an optimum quality of coexistence is one of the prime interests in education. For this reason, the goal of the present study was to focus mainly on the protective factors, such as quality of coexistence at school and social integration, measured by the perception of both positive and negative relationships among peers, related to diverse bullying forms: relational, aggressive and cyberbullying.

Our main findings emphasize that feeling socially integrated and perceiving few conflicts among students are important factors for each bullying type, although these factors are more relevant for relational bullying, which refers to the forms in which individuals are ignored, their participation in activities is neglected, or they are offended or humiliated. Although perceiving few conflicts was relevant for being bullied, perceiving positive relationships was not, which opens debate as to how to focus interventions, whether on promoting positive relationships or reducing conflicts, as they do not seem to be sides of the same coin.

With the correlation analysis, socio-demographic variables like gender or age were not relevant in our study sample to predict being bullied. We noted a greater and significant tendency toward aggressive bullying in boys than in girls. However this relationship was not strong. Regarding gender, mixed results were found based on bullying type (see Robers et al., 2014; Donoghue and Raia-Hawrylak, 2015; Innamorati et al., 2018). However, this gender difference depended on age, country or survey, as indicated by a recent review (Smith et al., 2018). So it is likely that no clear bullying pattern would emerge that could be differentiated by younger students. The remaining socio-demographic variables barely related directly with bullying. Instead, some were associated with number of good friends and social integration, such as the parents’ nationality and number of courses the student was enrolled for at the school. In particular, the students whose parents were both Spanish and those who had studied longer at the same school had more good friends and felt more integrated at school. Implications were of relevance because from educational and school viewpoints, schools should guarantee welcoming plans to ensure the integration of the new students who enroll at school (Mehta et al., 2013). Promotions to establish new friends and bonds should be provided, which would favor their social network and support, and would protect them from school victimization. This should be especially stressed for those cases in which children come from other countries or cultures, and for those whose enrollment at school takes place later than for other students (Valdivia et al., 2016). As previously reviewed, immigrant children may be a vulnerable minority for bullying (Llorent et al., 2016).

The subcomponents of quality of coexistence were strongly associated with each bullying type, as shown by the correlational analyses. Hence their role was confirmed by the mediational and SEM. Our findings revealed that the main predictor factors for all the bullying forms were number of good friends and school type. That is, the students with more good friends felt more integrated at school. The prediction of perceiving conflicts among students also lowered if the school was a state-funded school type. Several implications of these findings can be highlighted. Regarding school type, a previous study in Spain has suggested that some aggressive behavior types could be more prevalent in public schools, save some other forms related to verbal aggressive behavior, which was more commonly found in state-funded schools (Ruiz et al., 2014). In our study, only a direct relationship was found between school type and cyberbullying, although coefficient was low. Instead a relationship appeared between school type and perception of negative relationships and number of good friends, with them being lower in state-funded schools. Therefore, this finding is not conclusive as, while in public schools the number of friends is higher, also perception of relationships among peers is more negative. Nevertheless, as our sample was based mostly on public schools (almost 80%), no further interpretations could be made.

A greater sense of integration and low perception of conflicts were the main mediators for all bullying types, especially for relational bullying. This finding is consistent with previous findings which have emphasized the protective role of having friends, which lowers the likelihood of being bullied (Mucherah et al., 2018). As concluded in a recent systematic review and a meta-analysis, positive peer interaction is the strongest protective factor of being bullied (Zych et al., 2018). In particular in Spain, compared to other OECD countries, data are optimum as 87% of students in Spain feel integrated into their schools, unlike other OECD rates that indicated 73%. In addition, we add some pieces of evidence to support previous studies on the mediational role of the perception of relationships at school (Acosta et al., 2018). Interestingly, our findings indicated that positive relationships or cooperation relationships were not relevant against being victimized. Future prevention and intervention programs should focus not only on promoting cooperative relationships, but also on reducing conflicts, as this is a precursor for school bullying (Del Rey et al., 2018). Recent interventions with noteworthy effectiveness have more often included figures such as “peer mediators” or “peer supporters,” as well as “educator peers” which intervene when bullying is detected at school (Menesini and Salmivalli, 2017).

Both aggressive bullying and cyberbullying were very modestly predicted by the socio-demographic and quality of coexistence variables. These findings underline, on the one hand, that other factors are likely associated with these two other bullying forms, such as learning aggressive behaviors from parents (Griffin and Gross, 2004), or by personality traits related to aggressive behavior; e.g., impulsivity is one of the most well-studied (Carrera et al., 2011), or aggressive behavior may be a reaction triggered by some interpretation of others’ behaviors (Hanish and Guerra, 2000) rather than being related to school factors. On the other hand, the cyberbullying phenomenon is more likely to occur in students aged 10–11 years old, with a peak rate at 13–14 years (Sakellariou et al., 2012; Garaigordobil, 2015; Peris et al., 2018). Thus for our sample’s age range, this victimization could still be scarcely present. Moreover, some findings have indicated that cyberbullying is a form of violence that differs from the bullying which occurs at school and, therefore, from distinct predictor factors (Kubiszewski et al., 2015), in which some aspects like number of good friends, are relevant for relational bullying, which has very little to do with this cybernetic form of violence (Wang et al., 2009).

This study has several limitations. First, its design is cross-sectional and, therefore, no causality relationships can be established. Second, as the sample was recruited from a particular region of Spain, the generalizability of the results remains unknown. Third, data were collected from a self-reported measure, like most of the research based on this measurement type, with aspects such as social desirability, among others, which could bias our results. As the study formed part of a larger project, quality of coexistence and, in particular, all of its subcomponents, were evaluated by only a few items. Hence further research should more profoundly explore this construct with larger and more detailed questionnaires, accompanied by qualitative information. In addition, although this study also provides some psychometric properties of the used scales, which were not previously reported by the original authors, more in depth psychometric analyses should be conducted with further evidence for validity and invariance, among others, as some subscales yielded low reliability values; e.g., perception of positive relationships. Finally, and particularly in relation to how friendship was measured by number of good friends, although previous research has commonly used this indicator, a more in-depth analysis should be conducted in future studies to better know what “good friends” really means for students: for example, from sharing time together, sharing concerns or secrets, to displaying helping and supportive behaviors. Although most research agrees that number of good friends protects from bullying, sometimes victims make more friends with other victims, whereas perpetrators are friends who display other similar abusive behaviors (Salmivalli et al., 1997). Therefore, further research should explore the complex interaction and relationships among students more profoundly, which is a key factor to gain a better understanding of bullying.

Nonetheless, our study provides findings with direct implications for education for the bullying issue. In summary, these implications are related with having to promote integration among students at school as this emerged as the main protective factor from relational bullying, and to focus interventions to reduce conflicts among students. Finally, although most interventions tend to focus on secondary education, prevention should be addressed at earlier ages (Sánchez and Cerezo, 2011).

## Data Availability

The datasets generated for this study are available on request to the corresponding author.

## Author Contributions

NM wrote and performed the statistical analyses. EA and JC wrote the manuscript. JA performed the statistical analyses.

## Conflict of Interest Statement

The authors declare that the research was conducted in the absence of any commercial or financial relationships that could be construed as a potential conflict of interest.
